# Natural Compounds with Potential to Modulate Cancer Therapies and Self-Reactive Immune Cells

**DOI:** 10.3390/cancers12030673

**Published:** 2020-03-13

**Authors:** Rhiane Moody, Kirsty Wilson, Anthony Jaworowski, Magdalena Plebanski

**Affiliations:** School of Health and Biomedical Sciences, RMIT, Bundoora 3083, Australia; s3740510@student.rmit.edu.au (R.M.); kirsty.wilson2@rmit.edu.au (K.W.); anthony.jaworowski@rmit.edu.au (A.J.)

**Keywords:** self-reactive, natural compounds, immune response, ovarian cancer, native Australian plants, immunotherapy

## Abstract

Cancer-related deaths are approaching 10 million each year. Survival statistics for some cancers, such as ovarian cancer, have remained unchanged for decades, with women diagnosed at stage III or IV having over 80% chance of a lethal cancer recurrence after standard first-line treatment (reductive surgery and chemotherapy). New treatments and adjunct therapies are needed. In ovarian cancer, as in other cancers, the immune response, particularly cytotoxic (CD8^+^) T cells are correlated with a decreased risk of recurrence. As well as completely new antigen targets resulting from DNA mutations (neo-antigens), these T cells recognize cancer-associated overexpressed, re-expressed or modified self-proteins. However, there is concern that activation of self-reactive responses may also promote off-target pathology. This review considers the complex interplay between cancer-reactive and self-reactive immune cells and discusses the potential uses for various leading immunomodulatory compounds, derived from plant-based sources, as a cancer therapy option or to modulate potential autoimmune pathology. Along with reviewing well-studied compounds such as curcumin (from turmeric), epigallocatechin gallate (EGCG, from green tea) and resveratrol (from grapes and certain berries), it is proposed that compounds from novel sources, for example, native Australian plants, will provide a useful source for the fine modulation of cancer immunity in patients.

## 1. Introduction

The impact of cancer is seen globally, and it is one of the biggest burdens of disease, both in terms of morbidity and quality of life. In 2018, cancer accounted for approximately 9.6 million deaths worldwide [1]. The tumour microenvironment (TME) is an inflammatory environment, containing various inflammatory and regulatory mediators such as cytokines (tumour necrosis factor (TNF), Interleukin (IL)-1β, IL-6 and IL-10), as well as chemokines and reactive oxygen species (ROS) [2,3]. Many immune cell subsets are also present in the TME, creating complex interactions involved in the pro-tumour or anti-tumour response. Identifying and understanding these immune cells, and their specific targets, called antigens, support strategies to modulate the immune system and boost the anti-tumour response. One strategy to strengthen the anti-tumour response is by using immunotherapies—of which, the most prominent immunotherapy to date has been checkpoint inhibitors. Encouraging patient outcomes have already been observed in melanoma [4], non-small-cell lung carcinoma [5] and urothelial cancer [6] when modulating the immune response with T cell checkpoint inhibitors. T cell checkpoint inhibitors target programmed death-1 (PD-1)/programmed death ligand-1 (PD-L1) and cytotoxic T lymphocyte antigen 4 (CTLA-4), pathways that regulate immune responses by inhibiting T cell activity [7,8]. Checkpoint inhibitors are antibodies that block the “off” signal to T cells, allowing continued activation of T cells and killing of tumour cells. However, off-target side effects have been observed in ~ 70%–90% of patients using checkpoint inhibitors, depending on blockade treatment [9,10]. Therefore, novel supplementary therapies for cancer treatment are also being investigated, including those from natural sources, such as phenolic compounds.

### The Link between Immunotherapies, Adverse Events and Self-Reactive T Cells

Immune-related adverse events (irAEs) have been observed in patients undertaking checkpoint immunotherapies [11,12]. Not limited to particular cancer types, irAEs have been observed in patients with non-small-cell lung carcinoma [13,14], melanoma [15,16], ovarian cancer [17,18] as well as others. In each case, these typically present as dermatological, gastrointestinal and/or endocrine inflammatory side effects, e.g., dermatitis, colitis, and pancreatitis (reviewed by Trinh et al [9]) [19]. While the precise pathology of irAEs is unknown, possible mechanisms suggested by Postow et al [19] include an increase in T cells which are specific for antigens in both the tumour and healthy tissues, an increase in pre-existing autoantibodies, increased inflammatory mediators and enhanced complement mediated inflammation [19].

The potential of irAEs caused by T cells specific for antigens in both tumour and healthy tissue introduces a potential role of self-reactive immune cells. Tumour antigens which can activate the immune response can be cancer testis antigens, completely new antigens (neoantigens) or either overexpressed or slightly modified self-antigens [20,21,22]. Although found at high frequencies in healthy tissues [23], self-reactive T cells remain suppressed and their activation typically occurs when immune tolerance, the mechanisms which suppress immune responses to self-antigens, are dysregulated [24]. The activation of self-reactive cells is often associated with autoimmune diseases. However, reports have identified self-reactive cells to be immune regulatory, physiological (beneficial) or pathological (detrimental) (reviewed in [25]). In the context of cancer, the presence of increased levels of activated self-reactive immune cells has been identified and explored for potential diagnostic [26] and therapeutic [27] options. Self-reactive immune cells can be activated or exacerbated due to increased presence of their cognate antigen [28] and through inflammatory signals [29,30], which may explain the presence of increased self-reactive cells at diagnosis in cancer patients [31,32,33,34,35,36]. However, the exact role of these self-reactive cells is unknown, and further studies are required as to whether they would provide further therapeutic benefit if their anti-tumour response could be boosted.

While immunotherapies have shown benefits in some cancer types [4,5,6], others such as ovarian cancer still have high recurrence and mortality rates [37]. For this reason, there remains a drastic need for novel therapies. Self-reactive immune cells may provide targets for boosting the anti-tumour response. Indeed, it is observed in the clinics that patients who develop irAEs do have overall better clinical outcomes [38,39,40]. Here, irAEs are seen as an indicator of positive therapy response. However, understanding and controlling irAEs is also critical when considering self-reactive immune cells as therapy targets. Currently, corticosteroids are prescribed to combat irAEs [9]. However, in some cases, an additional immunomodulatory treatment is required [41], especially if there is a pause in the use of some immunotherapies, for example checkpoint inhibitors, [9,41] or in some cases discontinuation of those therapies. Therefore, other adjunct modulatory therapies may be considered to manage these off-target side effects, without the need to discontinue immunotherapies, or potentially as a possible replacement therapy.

A large body of research has focused on the potential use of natural compounds with anti-inflammatory and immunomodulatory properties, to complement standard cytoreductive surgery and chemotherapy/radiation treatment options. In this review, we will describe the immune system and potential utilities of self-reactive cells and outline the evidence for natural compounds as novel adjunct therapeutic options as well as potential alternatives to treating off-target effects.

## 2. Immune Cells in the TME

**Figure 1:**
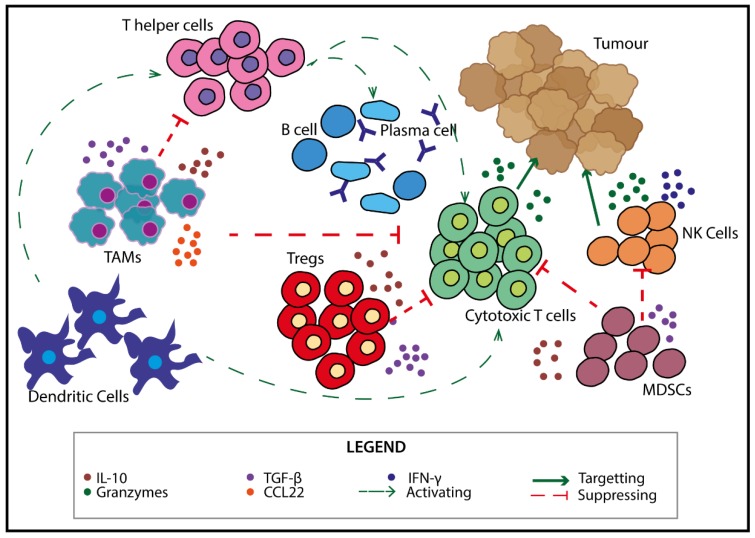
Immune cells in the tumour microenvironment (TME). There are multiple types of immune cells in the TME including dendritic cells (DCs), natural killer (NK) cells, myeloid-derived suppressor cells (MDSCs), tumour-associated macrophages (TAMs) and lymphocytes (B cells and T cells) which can impact the functions of one another. DCs interact with both T helper (Th) and cytotoxic T cells (CTLs) by activating them. The cells themselves can further interact with CTLs and B cells to boost activation. CTLs and NK cells directly act to kill tumour cells via the release of granzymes [47,48]. Pro-tumour cells such as regulatory T cells (Tregs), MDSCs and TAMs secrete immunosuppressive cytokines such as TGF-β and IL-10 which can inhibit CTLs and NK cell functions [49,50,51,52,53].

In the TME, cells of the immune system can play important roles in either anti-tumour or pro-tumour responses. The balance of immune cell subsets is correlated with tumour progression and recurrence in various cancers including, but not limited to, breast [42], lung [43] and ovarian [44,45,46] cancers. Many immune cell subsets exist in the TME, creating a diverse array of interactions and a complex balance between activation and suppression of the anti-tumour response (Figure 1).

The presence of activated, mature dendritic cells (CD107a^+^ DCs) in the ovarian cancer TME is correlated with a better prognosis [54]. They attract anti-tumour cell subsets (cytotoxic T lymphocytes (CTLs) and natural killer (NK) cells) but also pro-tumour cells (tumour-associated macrophages (TAMs), myeloid-derived suppressor cells (MDSCs) and regulatory T cells (Tregs)) to the TME. In ovarian cancer, an increased presence of anti-tumour CTLs and NK cells in the TME is associated with a favourable outcome [44,45,46,55]. T helper cells (Th), B cells and plasma cells are also found in the TME. The presence of B cells and antibody (Ab) producing plasma cells are found to be correlated with a better prognosis in ovarian cancer [56,57], suggesting that these cells assist in the anti-tumour response. However, their exact mechanism of function in the TME is unknown. Th cells assist B cells and CTLs to enhance the immune response against the tumour [58,59].

Tregs, MDSCs and TAMs are recruited into the TME by the tumour to counteract this anti-tumour response. These cells produce anti-inflammatory cytokines such as IL-10 and tumour growth factor (TGF)-β, which can suppress activation and cytotoxic function of CTLs and NK cells [49,50,51,52,53], as well as further induce Tregs [49]. The presence of Tregs as a ratio to CTLs can be used as a prognostic indicator [60]. In ovarian cancer patients, an accumulation of Tregs in the ascites (fluid buildup in the peritoneal cavity) typically indicates a more advanced disease, with a poorer prognosis [50]. Similarly, increased numbers of MDSCs and TAMs are correlated with poorer patient prognosis [61,62]. In the context of ovarian cancer, TAMs are one of the most abundant immune populations [63] and produce cytokines such as IL-10 and TGF-β, as well as the chemokine CCL22, which attracts Tregs to the TME [50], assisting in the pro-tumour microenvironment.

## 3. Self-Reactive Cells—Targets for Novel Therapeutics?

As suggested above, self-reactive cells could play a role in the pathogenesis of irAEs [19]. Indeed, by sequencing T cell receptors, case studies have identified overlapping T cell clones between tumour location and peripheral lesions which appeared following checkpoint inhibitor treatment [64,65]. These studies potentially indicate a role of the pathogenic type self-reactive cells in the irAEs, experienced due to checkpoint inhibitors. However, the fact that irAEs correlate with overall clinical outcome suggests that there is the potential that self-reactive immune cells may be important in the anti-tumour response. Furthermore, the increased presence of self-reactive cells, in both the TME and peripheral blood, has been assessed for both diagnostic and therapeutic value. Increased levels of antibodies specific to self-targets, known as autoantibodies (AAb), have been identified in several types of carcinomas including melanoma [31], lung [32], breast [33], gastrointestinal [34] and ovarian [35] carcinomas. In these cases, they have been explored for their diagnostic value. While explored as potential diagnostic biomarkers, the exact role of Ab and AAb in the TME remains unclear. In contrast, the presence of self-reactive T cells has been explored for their therapeutic value. Targets such as Her2/neu [66] and p53 [67], which are cell growth regulators and therefore self-antigens, have been found to be overexpressed in numerous cancer types. They have been used as candidates in vaccination strategies to boost levels of self-reactive T cells directed to cancer cells expressing these antigens.

### Utilising Self-Reactive T Cells to Modulate the Anti-Tumour Response

Studies have identified self-reactive T cells in cancer patients, and early phase clinical trials have used peptides derived from self-antigens as components of vaccinations aimed to induce potent T cell responses directly against the tumour [67,68]. P53 is overexpressed in many ovarian cancer patients and increased levels of AAbs recognising p53 have been reported [69]. Using IFN-γ ELISpot, memory T cells specific for p53-derived epitopes have been identified in ovarian cancer patients [36]. For this reason, peptides derived from p53 sequences have been proposed as antigens in a potential adjunct vaccination strategy to boost the anti-tumour immune response. Treatment with a p53 vaccine, in combination with gemcitabine chemotherapy, has shown benefits in chemotherapy (platinum) resistant patients [27]. In a small cohort, the expansion of anti-p53 specific CD4^+^ and CD8^+^ T cells resulted in a longer progression-free survival [27]. P53 vaccines in combination with chemotherapy have been shown to be beneficial in other cancers, such as small-cell lung cancer [67]. Another overexpressed protein identified in ovarian and breast cancer patients is HER2/*neu*. Using peptides specific for the HER2/*neu* protooncogene, CD8^+^ T cells were identified in both ovarian and breast cancer patients [70]. In further studies undertaken in ovarian cancer patients, in vitro cytotoxicity assays (chromium release assay) using a peptide from HER2/*neu* induced cytotoxicity against the autologous tumour [71]. In each of these cases, patient samples have been tested at the time of diagnosis, prior to treatment.

Early phase clinic trials have also been undertaken using vaccines targeting HER2/*neu* in ovarian and breast cancers [68,72]. In a separate phase I study, peptides targeting HER2/*neu,* human telomerase reverse transcriptase (hTERT) and pan-DR epitope (PADRE) were combined with dendritic cells, isolated from ovarian cancer patients, to be used as a vaccine [72]. Of 11 patients enrolled in the trial, 5 showed no evident disease in the follow up period, and 4 experienced disease recurrence. However, in each of these described cases of vaccine clinical trials, the women who took part had already undergone multiple rounds of chemotherapy. With promising results in these patients, identifying tumour-specific self-antigens associated with current first-line treatment may in fact provide vaccination targets to be used in conjunction with first-line therapy. However, there are still further studies required, and designing therapeutic vaccines faces a multitude of challenges. Mostly, these revolve around the facts that cancer antigens are not well defined, or derived from self-antigens, and the cancer TME is highly suppressive towards activating an immune response which requires potent adjuvant systems or cellular therapy to overcome this [22,73]. Therefore, other therapies for established cancers are being explored.

## 4. Treating Cancer and the High Risk of Recurrence

The standard treatment for many solid tumours, including ovarian cancer, is a cytoreductive surgery followed by first-line chemotherapy [74]. The type of surgery performed is largely determined by the spread of the mass [75]. Due to late diagnosis in most women, surgery often consists of a total hysterectomy (removal of uterus, cervix, fallopian tubes and ovaries) [75]. In more extreme cases, lymph nodes, parts of the bowel or other organs may be required to be removed [75]. In contrast, women who are diagnosed early may only need one ovary and fallopian tube removed [76].

Currently, first-line chemotherapy in ovarian cancer patients consists of a combination-based therapy with carboplatin and paclitaxel [74,77]. Up to 80% of patients initially respond well to the treatment, resulting in minimal residual tumour [78]. However, despite this initial response, those with advanced stage, metastasised tumours have an extremely high risk of recurrence (> 70%) [79], which has been attributed to the cancer stem cell (CSC) theory, in which CSCs survive during chemotherapy and are able to reinitiate tumour growth and metastasis [80,81]. This is not unique to ovarian cancer. Examples of other cancers with high recurrence rates include peripheral T cell lymphoma (75% [82]) and late stage melanoma (87% [83]). Some of these cancers, as well as others such as multiple myeloma, liver cancer and lung cancer, additionally have extremely low 5-year survival rates [84]. It is for this reason that novel options of therapies need to be explored or used in conjunction with immunotherapies or chemotherapy.

### Emerging Combination Therapies for Cancer

Immune interventions, such as checkpoint inhibitors, primarily aim to increase the cytotoxic ability of cells to directly kill the tumour. As previously mentioned, encouraging results have been seen in some cancer types following treatment with checkpoint inhibitors (e.g., melanoma and non-small-cell lung carcinoma) [4,5,6]. However, this is not the case for all cancers, including ovarian cancer, in which response rates remain poor (reviewed in [85,86]). Checkpoint inhibitors have been shown to be more effective in cancers with high somatic mutation burden [87,88,89]. Ovarian cancer, however, is not found to have a high burden of tumour mutations [90]. For this reason, exploring other emerging therapies as combination therapies may be beneficial.

Poly-ADP ribose polymerase (PARP) inhibitors are a potential therapy to be explored in such a way. PARP is a protein which assists in DNA repair. PARP inhibitors block DNA repair within tumour cells, leading to DNA damage and tumour cell death [91]. A combination of PARP inhibitors and checkpoint inhibitors in a small-cell lung carcinoma animal model showed greater potency than monotherapy [92]. Similar beneficial outcomes have been observed in ovarian cancer mouse models [93,94,95]. PARP inhibitors have additionally been used in multiple clinical trials (reviewed in [96]). PARP inhibitors have been found to induce IFN-α anti-tumour responses and promote tumour infiltrating cells which was enhanced by the checkpoint inhibitors [94]. Another mechanism in which PARP inhibitors and checkpoint inhibitors may work together is through the increased mutation burden, from DNA damage [91], which could modulate the TME to be more receptive to the anti-tumour immune response leading to beneficial outcomes of checkpoint inhibitor therapies.

Histone deacetylases (HDAC) inhibitors are another emerging therapy for cancer treatment. HDAC inhibitors act by inducing tumour cell cycle arrest and tumour cell death, reducing angiogenesis and by also modulating the immune response (reviewed in [97]). In an autoimmune disease, systemic lupus erythematosus (SLE), HDAC inhibitors were found to decrease the mRNA expression of inflammatory cytokines including IL-6, IL-12 and IFN-γ [98]. A decrease in IL-10 expression was also observed [98]. In cancer, major histocompatibility complex (MHC) and co-stimulatory molecule expression increases following treatment with HDAC inhibitors allowing for activation of T cells and increased susceptibility to NK cell killing [99,100]. Currently, HDAC inhibitors are primarily approved for patients with hematological cancers [97]. However, further studies are still required for solid tumours. A preliminary study in ovarian cancer cell lines showed HDAC inhibitors increased the sensitivity to cisplatin, therefore suggesting a combination of HDAC inhibitors and chemotherapy may be effective in targeting aggressive tumours [101]. For aggressive cancers and those with a high risk of recurrence, other novel compounds that modulate the immune system are also currently being investigated as potential adjunct therapy options, such as those derived from natural sources.

## 5. Natural Compounds as Anti-Cancer and Autoimmune Mediators

Natural compounds have been investigated for their effects on numerous cancers, including gynaecological malignancies [79], glioblastoma [102], pancreatic cancer [103], breast cancer [104,105], lung cancer [106], prostate cancer [107], colorectal cancer [108,109] and melanoma/skin cancers [110,111,112]. Compounds are mainly selected for their anti-inflammatory properties although evidence has now been obtained that numerous compounds may induce direct killing of cancer cells, i.e., by apoptosis, but also because they can mediate cellular mechanisms and signalling pathways [113]. Phenolic compounds can be extracted from many natural plant sources, mainly cereals, fruits, and vegetables, but also from fungi [114,115]. In a clinical trial of cervical, endometrial and ovarian cancer patients undergoing carboplatin chemotherapy, patients were also given oral mushroom extract (ABMK) for at least three cycles of chemotherapy. NK cell activity was increased in the ABMK treated patients, and negative side effects of chemotherapy were reduced [116]. These examples illustrate that supplementation with natural products can not only have anti-tumour effects but may also increase the quality of life by reducing side effects during chemotherapy.

The well-established anti-inflammatory and antioxidant activities of phenolic compounds make them suitable therapeutic options in other disease contexts, namely autoimmunity, especially if they can regulate self-reactive cells. In the context of autoimmunity, epigallocatechin gallate (EGCG, from green tea) has been shown to suppress self-reactive T cells and proinflammatory cytokines, though increase protective Treg responses in peripheral tissues in rheumatoid arthritis [117]. Similarly, EGCG contributed to reduced Th17 cell activation and increased FoxP3^+^ Treg cells in a model of autoimmune arthritis [118]. It is important to note that in the context of autoimmunity, Tregs are important for regulating autoreactive cells. However, in the context of cancers, locally induced Tregs in the TME are highly suppressive and contribute to disease progression. EGCG has also been reported to have cytotoxic activity for tumour cells and can attenuate molecular pathways involved in the progression of cancer [119]. This highlights the need for careful consideration of the compounds investigated in the context of different diseases, and how the modulation of immune cells is also directly related to other factors and off-target effects in the surrounding environment. For example, the presence of self-reactive immune cells in autoimmune diseases is detrimental, and dampening their response is preferential, though, in cancer, self-reactive cells may benefit the anti-tumour response. Phenolic compounds capable of modulating self-reactive cells may assist in regulating the potential off-target side effects of current immunotherapies.

### 5.1. Immune Cell Modulation by Natural Compounds

Though most natural compounds are initially investigated for their antioxidant and anti-inflammatory activity, the effects of natural compounds on the immune system is a growing area of interest. Not surprisingly, the compounds that have been examined for their immunomodulatory effects are those that have been well characterised and studied in various disease contexts (for example, inflammatory and autoimmune diseases, and cancer) such as resveratrol, curcumin and EGCG.

#### 5.1.1. Resveratrol

Resveratrol is found in some fruits, such as grapes, and berries (i.e., blueberries, cranberries and mulberries) and has been studied for its immunomodulatory and anti-cancer properties [120]. Resveratrol has been shown to increase apoptosis of human ovarian cancer cell lines (SKOV3) and administration of resveratrol decreased tumour progression in a murine in vivo ovarian cancer model due to increased CTLs and antigen presenting cells in the tumour tissue [79]. In the same study, decreased TGF-β and increased IFN-γ levels were observed in tumours in a murine model following resveratrol treatment [79]. Resveratrol has also been shown to induce apoptosis of leukemic cell lines [121], providing further evidence of its cytotoxic anti-cancer properties. Others have reported that resveratrol is able to directly promote an anti-tumour response by increasing NK cell killing of breast tumour cells [122]. Resveratrol is also able to modulate indoleamine 2,3 dioxygenase (IDO) expression which is important in the tumour microenvironment, as IDO expression on tumour cells contributes to immune evasion [123]. Resveratrol suppression of IDO may contribute to the decreased tumour progression, possibly by increasing immune recognition. An analog of resveratrol, HS-1793, increases IFN-γ production, resulting in decreased suppressive cytokine production, i.e., less IL-10 and TGF-β [124]. Resveratrol has been reported to disrupt the progression of lung metastasis by decreasing the number of regulatory B cells (Bregs) via the inhibition of the Stat 3 pathway [125]. Other studies have also shown decreases in Tregs and TGF-β production in animal models of cancer treated with resveratrol, along with increases in IFN-γ production in CD8^+^ T cells [126].

#### 5.1.2. Curcumin

Curcumin, from turmeric, is another compound that has been extensively studied for its immunomodulatory and anti-cancer properties [127,128]. In studies where model antigens have been administered in animal models, it has been reported that curcumin supplementation increased T follicular helper cells and germinal centre B cells as well as subsequent IgG production [129]. The data on effects of polyphenols on immune cells is increasing, especially in the context of cancer [130], with a lot of research understandably focusing on the effects on Tregs [131]. Modulating this cell type is especially important for ovarian cancer [132,133,134]. Curcumin can modulate Treg numbers and FoxP3 expression [135]. Decreased numbers of Tregs and increased Th1 cells have been reported in lung cancer patients treated with curcumin [136]. Similar results were seen in colon cancer patients, where curcumin treatment decreased the total number of Tregs and expression levels of FoxP3 as well as increased Th1 cells [137]. In an animal model of tongue squamous cell carcinoma, curcumin decreased PDL-1 expression on the oral adenocarcinoma cell lines in vitro and additionally reduced tumour growth and stimulated the anti-tumour immune response by decreased numbers of Tregs and MDSCs in vivo [138]. In addition to suppression of Treg function, curcumin was able to prevent the depletion of T cells by tumours and promoted the expansion of central and effector memory T cell phenotypes [139]. The ability of this compound to decrease suppressive cells and retain lymphocyte numbers, especially CD8^+^ T cells, in the TME is important in the context of the anti-tumour immune response.

#### 5.1.3. EGCG 

The polyphenol EGCG is another compound known for its anti-cancer and immunomodulatory properties. EGCG reduced Treg numbers and lymphocytosis in a small clinical trial of chronic lymphocytic leukemia (CLL) [140]. EGCG was similarly able to induce apoptosis and increase the expression of the apoptosis inducer proteins Bax and caspase 3 in esophageal squamous cell carcinoma cells [141], and induced apoptosis in hepatocellular carcinoma cell lines [142]. Furthermore, EGCG induced apoptosis and cell cycle arrest in ovarian cancer cell lines, inhibiting cancer cell growth in vitro [143,144]. Derivatives of EGCG induced apoptosis and inhibited proliferation of non-small-lung-cancer cell lines, especially in combination with the chemotherapeutic cisplatin [106]. EGCG has additionally shown synergistic activity with other anti-cancer treatments, such as tamoxifen (hormone therapy) and nonsteroidal anti-inflammatory drugs (NSAIDs, i.e., cyclooxygenase-2 (COX-2) inhibitors) [145]. EGCG and whole green tea extract also acted as immune checkpoint inhibitors by decreasing the expression of PDL-1 on alveolar adenocarcinoma cell lines and in in vivo murine lung cancer models [146]. It is known that EGCG affects signalling pathways involved in proliferation and apoptosis and is able to inhibit nuclear factor kappa-light-chain-enhancer of activated B cells (NFκB) pathways and other molecular targets involved in the progression of cancer [119,147]. EGCG also alters epigenetic processes [148] through interaction with DNA methyltransferases and histone deacetylases, though this is affected by its low bioavailability.

Aside from the direct apoptotic inducing effects of EGCG, the effects on immune cells have also been investigated. EGCG can inhibit TAM infiltration into subcutaneously implanted breast cancer tumour cells in murine models, potentially via upregulating microRNAs in tumour cells which were subsequently released via exosomes to inhibit the recruitment of TAMs [149]. Though there are some positive reports of EGCG and immune modulation relating to anti-cancer responses, studies have shown a decrease in Th1 and CD8^+^ T cells and an increase in Tregs after administration of EGCG [150]. Therefore, EGCG may be beneficial in supplementing treatment for autoimmune diseases, rather than cancers that require a strong CTL response and reduced Tregs, such as ovarian cancer. On the other hand, this compound may be suited to modulate the off-target effects of self-reactive cells in the TME, and it would be of interest to determine the effects of EGCG in combination with checkpoint inhibitors, especially in the context of irAEs.

### 5.2. Bioavailability of Natural Compounds

Polyphenols have limited bioavailability, which restricts their application and translatability. Possible solutions to increase the bioavailability of these compounds are to attach them to particulate carriers or use them with another stimulant/adjuvant to increase their recognition. Increasing the bioavailability of natural compounds increases their effectiveness against cancer cells. For example, Tricurin (combination of curcumin, EGCG and resveratrol) formulated in liposomes increased apoptosis of glioblastoma cells in vitro and in vivo, and modulated the phenotype of microglia to upregulate their anti-tumour activity [151].

To increase the bioavailability of curcumin, studies have shown that ligating curcumin with polyetyhyleneglycol (PEG) resulted in increased CD8^+^ T cell activity and effector cytokine (IFN-γ) production, as well as decreased MDSCs and Treg concentrations in an animal model of melanoma [152]. In another study, a synthetic analogue of curcumin, hydrazinocurcumin, encapsulated in liposomal nanoparticles, changed the phenotype of TAMs to anti-tumourigenic cells capable of inhibiting primary tumour and metastatic growth in a breast cancer model, especially when ligand targeting nanoparticles were used [153]. The increased activity of hydrazinocurcumin is attributed to its ability as a STAT3 phosphorylation inhibitor, leading to suppression of proliferation in tumour cell lines and contributing to the phenotypic modulation of TAMs [153,154]. Alternative approaches to increase the bioavailability of curcumin have also been studied. Curcumin complexed with different metal ions has been tested for antibacterial inhibition activity, though only curcumin complexed with cobalt displayed antibacterial properties against *Bacillus subtilis, Escherichia coli, Pseudomonas aeruginosa* and *Staphylococcus aureus* [155].

Various nanoformulations have been used to increase the bioavailability of different polyphenols [156]. The advantage of nanoparticle carriers is that they can target the tumour, have increased stability and the polyphenol can be associated with the particle in various ways. Direct attachment to the surface, encapsulation of the polyphenol (with the option of co-encapsulation with other compounds), or incorporation within the surface of the nanoparticle are examples of methods used to associate nanoparticles with polyphenols. Nanoparticles have been used to increase the efficacy of EGCG as a targeted cancer therapeutic [157,158]. EGCG compounds alone can induce apoptosis and inhibit proliferation of cancer cells, although sometimes at doses that are not physiologically possible. Encapsulating EGCG in chitosan nanoparticles drastically reduced the amount of EGCG required to inhibit growth of melanoma cell lines and induce pro-apoptotic proteins, resulting in an approximately 8–10 fold decrease in effective doses [159]. Bovine serum albumin nanoparticles formulated with resveratrol have also been shown to induce apoptosis of human ovarian cancer cell lines in a caspase-dependent manner, which occurred via a different mechanism to the pro-apoptotic effects of non-nanoparticle-encapsulated resveratrol [160]. Utilising nanoformulations in combination with polyphenols is one technique that could be applied to other identified novel compounds with limited bioavailability.

## 6. Natural Compounds from Native Australian Plants

Many of the known phytochemicals have been sourced from plants, herbs and spices obtained from various geographical locations, though there are many more yet to be tested for their anti-cancer and immunomodulatory properties. Novel natural compounds which may be examined for potential anti-tumour properties have also been identified in native Australian plants (Table 1). For example, *Scaevola spinescens* is a native Australian shrub that has been documented in medicinal preparations by indigenous Australians, and studies of extracts from this plant have shown them to be non-toxic and demonstrate antibacterial and antiviral activity [161]. High-Performance Liquid Chromatography (HPLC) and mass spectrometric analysis of phenols present in native Australian mints has identified multiple phenolic compounds, with the antioxidant capacity of native mint comparable to extracts from other mint types (i.e., spearmint) [162]. Of the native Australian fruits researched in one study, Kakadu plum had high antioxidant levels and phytochemical content, as well as mineral levels comparable to other Australian and non-Australian fruits [163].

### 6.1. Phenolic Content of Native Australian Plants

Numerous native Australian plants and/or fruits have been screened for their antioxidant potential and phenolic content [164]. The polyphenols identified have mostly been studied for their antioxidant and anti-inflammatory properties, though some have begun to be examined for their therapeutic potential in certain diseases, including autoimmune diseases and cancers. Methanol and water extracts of the Tasmanian pepperberry have been shown to inhibit the bacteria identified as a trigger of autoimmune inflammatory disorders, such as rheumatoid arthritis [169]. Tasmanian pepper leaf and anise myrtle have been identified to have high phenolic content, including phenolic acids and flavonoids [167]. Lemon myrtle and Kakadu plum contain the flavanol catechin, and Kakadu plum, Davidson plum and wattle seed all contained the flavonone naringenin [164].

### 6.2. Therapeutic Activity of Whole Extracts from Native Australian Plants

Both phenolic compounds and whole extracts from native Australian fruits have been reviewed for their potential as anti-pancreatic cancer therapeutics [103], with a lot of this data applicable to other cancer types, including ovarian cancer. It has been shown that polyphenolic extracts (from acidified methanol, then purified and lyophilised) from native Australian fruits such as Illawarra plum, Kakadu plum, muntries (native cranberry or emu apple) and native current can inhibit the growth of cancer cell lines, inducing apoptosis pathways in leukemia cells [165]. Alcohol extracts from Tasmanian pepper leaf, anise myrtle and lemon myrtle decreased the proliferation of in vitro cancer cell lines for colon, stomach, bladder and liver cancer cells, and all extracts increased the apoptosis of leukemic cell lines [170]. In both instances, anise myrtle extract showed the highest anti-proliferative and pro-apoptotic capacity.

An acidified ethanol extracted fraction from Kakadu plum, termed KPF5, displayed anti-inflammatory activity by modulating inflammatory enzymes and inducing apoptosis in adenocarcinoma cell lines [171]. The polyphenols identified in the extract were novel and not known common polyphenols. Methanol extracted fractions from Illawarra plum also displayed anti-inflammatory activity, consistent with other reports of Illawarra plum extract decreasing the proliferation and increasing apoptosis of colon adenocarcinoma cells [172]. Anise myrtle, lemon myrtle and Tasmanian pepper leaf all have anti-inflammatory potential, inhibiting pro-inflammatory enzymes in a lipopolysaccharide (LPS)-activated macrophage in vitro model, with anise myrtle and lemon myrtle being more inhibitory, therefore potentially more anti-inflammatory, than Tasmanian pepper leaf [168]. Using the same LPS-activated macrophage model, it was shown that Kakadu plum, Illawara plum and native current, but not muntries, are able to inhibit inflammatory enzymes (i.e., iNOS and COX2), with Kakadu plum affecting the NFκB but not mitogen-activated protein kinase (MAPK) intracellular pathways [173] (phytochemical effects on molecular pathways reviewed in [174]).

While there is a substantial body of knowledge on the immunomodulatory properties of some specific natural compounds (such as curcumin and resveratrol), much less is known about the potential immunomodulatory properties of compounds derived from native Australian flora, either as whole extracts or the polyphenols thus far identified within them. The bioavailability of some of these compounds is also currently unclear, and it can only be speculated whether it could be improved via their formulation with nanoparticles or whether they could act synergistically with other adjuvants or therapeutic compounds.

## 7. Therapeutic Benefits of Isolated Phenolic Compounds

### 7.1. Hesperetin

Of the phenolic compounds identified from some of the above plants, plus other plant sources, a number have been reviewed for their therapeutic benefit. Hesperetin is a flavonone that is commonly found in some citrus, such as lemons and oranges, and has both antioxidant and anti-inflammatory properties [175]. Hesperetin derivatives have been shown to induce apoptosis in colon cancer cell lines [176], similarly, apoptosis and ROS accumulation were observed in breast cancer cell lines following hesperetin treatment [177]. In an eosophogeal cancer cell line, hesperetin induced apoptosis with increasing doses and increased the level of apoptosis inducer proteins Bax, caspase 3 and caspase 9 [178]. In the same study, tumour growth was inhibited in a murine xenograft tumour model in a dose dependent manner [178]. Hesperetin is reported to be hydrophilic, however, incorporating it into liposomes increased its stability and retained its anti-cancer properties against both lung and breast cancer cell lines [179].

### 7.2. Myricetin

Myricetin is a flavonoid that can be found in numerous vegetables, fruits and berries and is also known for its antioxidant and anti-inflammatory capacity. Its structure is related to that of quercetin and kaempferol [180]. Myricetin was shown to inhibit the proliferation of colon carcinoma cells [181], suppress extracellular signal-regulated (ERK) kinase phosphorylation and matrix metallopeptidase (MMP) enzyme activity and prevent invasion of colon cancer cells in a transwell migration assay [182]. Myricetin inhibited the growth of breast cancer cells to a greater extent than resveratrol and cisplatin and induced intracellular ROS and apoptosis in these cells [183]. Although myricetin demonstrates anti-cancer activity, the immunomodulatory effects of myricetin have mostly been studied in the context of dampening the T cell response as a possible autoimmune/autoreactive cell therapeutic. It has been shown to inhibit murine T cell proliferation and decrease IFN-γ, IL-2 and IL-17 secretion, attributed to the induction of ROS by myricetin [184]. Others have also shown that myricetin decreased IL-2 production in phorbol myristyl acetate (PMA)-activated T cells [185], and reduced IL-12 production in macrophages stimulated with LPS, as a consequence of decreased NFκB activation [186]. In dendritic cells stimulated by LPS, myricetin has been shown to decrease expression of TNF, IL-6 and IL-12p70, and decrease the activation markers CD40 and CD86, again attributed to downregulation of the NFκB and MAPK pathways [187]. The immunomodulatory effects of myricetin in the context of cancer immunity in vivo is yet to be determined, though the ability to decrease suppressive cytokines and modulate self-reactive cells is promising. 

### 7.3. Quercetin

Quercetin is another flavonoid commonly found in berries, onions and leafy vegetables that has anti-cancer properties [188]. Quercetin was able to reduce tumour burden in a in vivo colorectal cancer murine model [189] and also a murine lung tumour model [190]. Quercetin induced apoptosis and upregulated caspase 3 and caspase 9 in a malignant glioma cell line and induced an autophagy response in these cells [191]. In a hepatocellular cell line, quercetin increased the pro-apoptotic protein Bax and decreased signalling in the pro-survival PI3K pathway [192]. Similarly, in a breast cancer cell line, quercetin inhibited proliferation and upregulated Bax, leading to apoptosis in these cells [193]. It has been suggested that quercetin is immune-stimulatory, inducing IFN-γ production in peripheral blood mononuclear cells (PBMCs) [194]. It may also modulate inflammation as it prevents IL-1β-induced secretion of IL-6 from mast cells [195]. In an LPS-activated dendritic cell model, quercetin modulated DC activation and reduced secretion of TNF, IL-1β, IL-6, IL-10 and IL-12p70, [196]. Properties of quercetin, and other identified compounds, that reduce IL-6 and IL-10 show promise as a potential anti-cancer therapeutic, though reductions in TNF and IL-12 may not be favourable for anti-tumour activity as these cytokines can promote the anti-tumour response [197,198]. However, systemic activation of TNF (and IL-12) outside the TME can lead to adverse inflammatory responses and off-target effects of TNF therapy [199,200]. It would be of interest to investigate whether quercetin may be able to dampen the systemic off-target effects whilst retaining direct anti-tumour activity. Additional studies on the direct anti-tumour properties and immunomodulation in the TME of quercetin and other compounds with similar effects would be required to address these considerations.

### 7.4. Cyanidin-3-glucoside

Cyanidin-3-glucoside (C3G), found in blueberries, acai and black soybean, has exhibited antioxidant activity in epidermal cell lines by scavenging free oxygen radicals, as well as reducing NFκB and MAPK signalling [201]. In the same study, C3G prevented tumourigenic cell transformation and reduced the tumour burden in vivo in murine studies of induced skin tumours and also xenograft implanted human lung carcinoma cells [201]. Whilst there is little information on its anti-tumour immunomodulatory effects, studies have shown that C3G reduced rheumatoid arthritis presentation, and concurrently increased IL-10, Tregs, and decreased IL-6, IFN-γ and NK cell activity [202]. The potential anti-cancer properties of C3G, coupled with autoimmune modulation, may provide benefit for off-target effects of self-reactive cells in the TME.

## 8. Future of Natural Compounds as Potential Anti-Cancer Therapeutics

An alternative potential adjunct therapy for cancer is to supplement treatment schedules with natural compounds in the form of whole extracts or purified phytochemicals. Phytochemicals, chemical compounds produced by plants, have long been investigated for their therapeutic potential for a range of diseases, for example inflammatory and autoimmune diseases, and even as anti-cancer agents [203]. One of the main categories of phytochemicals under examination are polyphenols. Polyphenols have antioxidant and anti-inflammatory properties and can modulate autophagy pathways as well as modulate cells of the immune system [113]. It is likely that polyphenols modulate their anti-inflammatory effects via epigenetic mechanisms, such as DNA methylation and histone modification [204].

Specifically of interest to an anti-cancer response, especially for ovarian cancer, is the ability of certain polyphenols to modulate CTLs and NK cells, whilst concurrently dampening the Treg and MDSC responses. Some of the most extensively studied and characterised polyphenols include curcumin, resveratrol and EGCG. Of all the natural compounds that have been studied for their anti-cancer properties, curcumin and resveratrol have the most extensive evidence supporting both direct and indirect anti-cancer properties, as well as an ability to modulate immune cells in the TME [205,206]. There have also been studies on the synergistic effects of these compounds combined with EGCG, in a preparation termed Tricurin [207]. Still, there are many more compounds yet to be tested for their anti-cancer and immunomodulatory properties, such as those from native Australian plants. 

As with any therapeutic agent, dosage and possible side effects must always be taken into consideration. Some phytochemicals may be toxic at high doses but beneficial at low doses, with antioxidant, anti-inflammatory and anti-tumour effects [208]. For example, whole extracts of Kakadu plum contain beneficial phenols, such as ellagic acid, though there are also detrimental levels of specific compounds at high doses, such as oxalic acid which can bind calcium, preventing absorption, and can aid in the production of kidney stones [209]. Methods to increase bioavailability, such as using carrier systems, will also allow for decreased amounts of the polyphenols required to be used that still display a therapeutic effect.

A clear need is for many of these identified, and even unidentified, polyphenols to undergo testing for their immunomodulatory capacity. It will also be of interest in future to test whether isolated purified compounds have similar benefits as whole extracts, or whether there is a level of synergism between phenolic compounds present in extracts. For beneficial effects in the context of cancer, compounds may be required that have both apoptotic capacity and increase the cytotoxic and effector activity of T cells and NK cells whilst concomitantly negating off-target side effects of any combination therapies.

## 9. Conclusions

The presence and relative importance of self-reactive immune cells in the context of cancer remain understudied. Clinical studies have identified benefits of their presence in both diagnostic and therapeutic contexts. However, the presentation of irAEs, following treatment with checkpoint immunotherapies, is a less desirable outcome. Natural compounds may therefore assist in providing anti-inflammatory, immunomodulatory and anti-tumour effects, which may be able to modulate off-target side effects. Additionally, the development of irAEs has been associated with a better overall response. Therefore, careful considerations are required in order to maintain the beneficial outcome of checkpoint inhibition whilst removing undesirable side effects. Given these considerations, it is expected that future studies combining current immunotherapies and novel natural compounds will receive increased interest. Natural compounds, as a novel adjunct immunotherapy option, may give insights into ways to aid in the anti-cancer immune response, though much more evidence for this is needed. Future research in this field could ultimately provide additional therapeutic options for cancer treatments, especially to improve outcomes in aggressive and high mortality rate cancers, such as ovarian cancer.

## Figures and Tables

**Table 1:** Phenolic compounds from native Australian plants.

Common Name	Botanical Name	Known Therapeutic Potential	Phenolic Compounds Identified	Refs
Kakadu plum	*Terminalia ferdinandiana*	Antioxidant and induces apoptosis and inhibits proliferation in cancer cell lines	Catechin Naringenin Quercetin/hesperitin glucosides Kaempferol/luteolin glycosides	[164,165]
Illawara plum	*Podocarpus elatus*	Antioxidant and induces apoptosis and inhibits proliferation in cancer cell lines	Cyanidin 3-glucoside pelargonidin 3-glucoside	[165,166]
Davidson’s plum	*Davidsonia pruriens*	Antioxidant	Naringenin Hesperetin delphinidin 3-sambubioside cyanidin 3-sambubioside peonidin 3-sambubioside petunidin 3-sambubioside	[164,166]
Native river mint	*Mentha australis*	Antioxidant	Neoponcirin Rosmarinic acid Narirutin Chlorogenic acid Biochanin A	[162]
Muntries	*Kunzea pomifera*	Antioxidant and induces apoptosis and inhibits proliferation in cancer cell lines	Delphinidin 3-glucoside cyanidin 3-glucoside	[165,166]
Tasmanian pepper berry	*Tasmannia lanceolata*	Antioxidant	Cyanidin 3-rutinoside Cyanidin 3-glucoside Rutin Chlorogenic acid Caffeic acid Quercetin	[166,167]
Tasmanian pepper leaf	*Tasmannia lanceolata*	Antioxidant	Chlorogenic acid Quercetin p-Coumaric acid Cyanidin 3-glucoside	[167]
Anise myrtle	*Syzygium anisatum*	Antioxidant and anti-inflammatory	Chlorogenic acid Myricetin Quercetin Quercetin pentoside Ellagic acid Ellagic acid derivatives Catechin Hesperetin	[167,168]
Lemon myrtle	*Backhousia citriodora*	Antioxidant and anti-inflammatory	Catechin Epicatechin Vanilic acid Myricetin Hesperetin rhamnoside Hesperetin hexoside Quercetin Ellagic acid Ellagic acid derivatives	[164,167,168]
